# New-Onset Hyperreligiosity, Demonic Hallucinations, and Apocalyptic Delusions following COVID-19 Infection

**DOI:** 10.1155/2023/9792099

**Published:** 2023-02-11

**Authors:** Joseph Ahearn, Maggie Driscoll, Sahiti Gilela

**Affiliations:** ^1^Department of Psychiatry, Lehigh Valley Health Network, Bethlehem, Pennsylvania, USA; ^2^University of South Florida Morsani College of Medicine, USA

## Abstract

**Background:**

Neuropsychiatric sequelae of COVID-19 have been documented, including delusions, hallucinations, agitation, and disorganized behavior. Although the mechanisms for these symptoms remain unclear, there has been an increasing body of literature suggesting a correlation between COVID-19 infection and psychosis. Here, we illustrate the case of a 34-year-old female with no previous psychiatric history who contracted COVID-19 and subsequently developed severe symptoms of psychosis. After presenting to the emergency department with one month of worsening mood, auditory hallucinations, intrusive thoughts, and hyperreligiosity, she was admitted to the inpatient psychiatric unit. The patient was treated with multiple antipsychotic medications and was discharged in stable condition with resolution of her auditory hallucinations; however, her delusions, hyperreligiosity, and negative psychotic symptoms persisted, resulting in a second inpatient psychiatric admission eight days after discharge, during which she again did not reach full remission.

**Objectives:**

With this information, we hope to increase awareness of COVID-induced psychosis and further discuss the relationship between COVID-19 infection and neuropsychiatric symptoms.

**Conclusions:**

Although there has been increasing research about the COVID-19 pandemic, there is much to be elucidated regarding the neuropsychiatric symptoms related to these infections. Similar to previous studies, our case describes a patient with no previous psychiatric history who developed severe psychotic symptoms after COVID-19 infection and was admitted to the inpatient psychiatric unit. These symptoms resulting from infection can be severe or debilitating for the patient. Therefore, physicians should be aware of these potential neuropsychiatric sequelae when treating patients with active COVID-19 infections, and treatment with antipsychotics or acute inpatient psychiatric admission should be considered.

## 1. Introduction

Since 2019, the novel coronavirus (COVID-19 or SARS-CoV-2) has spread across the world, and there is still much to be known about this virus that caused a global pandemic. An increasing body of evidence suggests that mental health illnesses are sequelae of COVID-19 infections. Often this is due to indirect factors, such as financial hardships, isolation from one's support system, or fear of death. There is, however, increasing evidence that mental illness caused by COVID-19 is due to direct interaction with the central nervous system. A growing amount of literature suggests that new-onset psychosis is a possible psychiatric symptom of COVID-19. Multiple case reports have found a relationship between COVID infection and psychotic features, including persecutory delusions, agitation, and auditory and visual hallucinations [1–3]. One literature review included 42 cases and another described 48 cases of psychosis reported in infected patients [4, 5]. These patients also had similar symptoms of hallucinations, insomnia, persecutory delusions, and paranoia [4, 5]. We present the case of a 34-year-old female with no previous psychiatric history who developed severe psychosis with intrusive thoughts and hyperreligiosity after a case of COVID-19 infection.

## 2. Patient Information

Ms. T is a 34-year-old female with no significant past psychiatric history. She presented to the emergency department due to one month of worsening mood, inability to care for herself, hyperreligiosity, auditory hallucinations, and perseveration on the idea that she is going to hell. The patient reported auditory hallucinations in which she heard the voices of Satan or Saints. She reported anergia, amotivation, anhedonia, decreased appetite, and difficulty falling and staying asleep. The patient also had new-onset superficial cutting of her wrists. She was perseverating on the idea that she had become an apostate and was therefore going to hell. The patient denied suicidal ideation, homicidal ideation, or visual hallucinations. The patient's family history was relevant for a maternal grandmother with schizophrenia. After evaluation in the emergency department, acute inpatient psychiatric hospitalization was recommended. Ms. T signed herself into the psychiatric hospital voluntarily for her first hospitalization. The patient's parents reported that this behavior was unusual and abrupt and that she was previously sociable and well-liked by her peers. The patient's symptoms started within 3 weeks of the patient contracting COVID-19 which was confirmed via PCR. They deny any similar symptoms or similar behavior in the past. Her parents report that the patient had taken a leave of absence from work as she was frequently calling them from work crying. They report that the patient's speech was childlike, as if she had regressed.

## 3. Clinical Findings

Throughout the hospitalization, the patient exhibited flat affect, a significant response lag, thought blocking, and disorganized thought, often starting sentences without finishing them. The patient would frequently answer questions with “I don't know” or “I'm fine”. She appeared internally preoccupied. The patient was perseverating on the idea that the world was ending and that events which had been predicted in the Bible had come true, though she was unable to elaborate. The patient did attend group therapy sessions during the hospitalization but did not participate in group discussion.

## 4. Diagnostic Assessment

The patient was diagnosed with major depressive disorder, recurrent, severe with psychotic features. The differential diagnosis included schizoaffective disorder, depressed type, and schizophrenia. While the patient's diagnosis carries a better prognosis than schizoaffective disorder or schizophrenia, as is often the case with initial psychotic episodes, a longitudinal assessment would be necessary to determine the patient's diagnosis with greater certainty.

## 5. Therapeutic Intervention

Ms. T was initially started on aripiprazole which was titrated up to 10 mg daily. The patient showed little improvement and was transitioned to olanzapine which was titrated up to 20 mg nightly. The patient reported resolution of her auditory hallucinations; however, her delusions, hyperreligiosity, and flat affect persisted. The patient was discharged with planned follow-up with a partial hospitalization program. Unfortunately, she attended this program for only one half day before withdrawing from care against medical advice. Eight days later, the patient was voluntarily readmitted for acute inpatient psychiatric hospitalization for worsening delusions, worsening mood, and inability to care for herself. The patient was started on venlafaxine which was titrated up to 150 mg daily, and the olanzapine was continued. The patient's mood improved, and her delusions of going to hell resolved with the addition of the antidepressant; however, her negative symptoms including flat affect, withdrawal from social situations, apathy, and anhedonia persisted.

## 6. Follow-Up and Outcomes

The patient was discharged on day eight of the subsequent hospitalization with a plan for outpatient psychiatric follow-up. The patient agreed to attend these appointments; however, she stated repeatedly during the hospitalization that she does not have a psychiatric illness, does not need psychotropic medications, and merely needs to see a Christian counselor. Unfortunately, the patient did not attend her scheduled outpatient appointment and did not pursue further treatment.

## 7. Discussion

There is a small but growing database of evidence indicating that SARS-CoV-2 has the potential to induce neuropsychiatric symptoms during infection, including depression, anxiety, trauma-related disorders, demyelinating and neuromuscular complications, neurodegenerative disorders, and psychosis [6, 7]. Potential mechanisms that have been suggested include viral infiltration of the central nervous system, disruption of the cytokine network, peripheral myeloid cell recruitment to the CNS, autoimmune response [8], effects of concomitant corticosteroid treatment [9], or changes to gut microbial composition [10]. The virus is postulated to enter the central nervous system either hematogenously or via retrograde axonal transport of peripheral neurons after binding to angiotensin-converting enzyme 2 [11].

Psychosis secondary to a viral infection is by no means a novel process. A 2004 study identifies 15 patients without past psychiatric history who developed psychosis after infection with the 2003 SARS virus [12]. In this study, a family history of psychosis was more common in patients with SARS-induced psychosis than control subjects, consistent with our patient's family history of a grandmother with schizophrenia. Other cases of COVID-19-induced psychosis have identified a family history of mental illness other than primary psychotic disorders, such as bipolar disorder. It has also been shown that children born during the 1957 type A2 influenza epidemic exhibited an increased rate of developing adult-onset schizophrenia [13]. Of note, other mental disorders have been associated with both COVID-19 infections and SARS infections [14], including anxiety, mania, insomnia, memory loss, and depression [11]. One confounding factor for clinicians to be aware of is the frequent treatment of COVID-19 with steroids, which can cause steroid-induced psychosis. This does not, however, appear to be the sole factor causing psychosis in the wake of COVID-19 infections [12]. It is also vital for physicians to rule out delirium as this is common in patients with COVID-19 infections.

A retrospective chart review showed a 10% increase in diagnosis of new-onset psychosis in January 2020 compared to data from 2017 to 2019, although this increase could, at least in part, be attributed to increased stress caused by the pandemic [15]. Delusions have been found to be the most common symptom of COVID-19-related psychosis [5]. Of note, our patient presented with a primary psychotic content of delusions. Cases of hyperreligiosity following COVID-19 infections are rare but have been documented [16]. One systematic review found that 92% of cases of COVID-induced psychosis presented with delusions [5]. Other common symptoms include auditory hallucinations (60%) and disorganized behavior (48%) [5].

While the relevant literature suggests that the typical course of psychotic symptoms is relatively short with most cases exhibiting resolution of symptoms within two weeks [5], our patient did not achieve complete remission of her symptoms despite two lengthy hospital admissions. Further research is therefore needed to fully understand the mechanism by which COVID-19 causes neuropsychiatric sequelae and the specific treatment regimens that should be employed for such cases.
